# Changes in the components of visual attention following traumatic brain injury: A systematic review and meta-analysis

**DOI:** 10.1371/journal.pone.0268951

**Published:** 2022-06-09

**Authors:** Mohammed M. Alnawmasi, Revathy Mani, Sieu K. Khuu

**Affiliations:** 1 School of Optometry and Vision Science, The University of New South Wales, Sydney, Australia; 2 Department of Optometry, College of Applied Medical Science, Qassim University, Buraydah, Saudi Arabia; University of Essex, UNITED KINGDOM

## Abstract

**Purpose:**

We conducted a systematic review and meta-analysis to understand the impact of traumatic brain injury (TBI) on visual attention and whether different components and processes of visual attention (such as selective, sustained, divided, and covert orientation of visual attention) are affected following brain injury.

**Methods:**

A literature search between January 1980 to May 2021 was conducted using Medline, Scopus, PubMed, and Google Scholar databases was undertaken for studies that assessed visual attention using different tasks that target specific or multiple components of visual attention. Three hundred twenty-nine potentially relevant articles were identified, and 20 studies met our inclusion criteria.

**Results:**

A total of 123 effect sizes (ES) were estimated from 20 studies that included 519 patients with TBI and 530 normal participants. The overall combined ES was statistically significant and large (ES = 0.92), but with high heterogeneity (Q = 614.83, p < 0.0001, I^2^ = 80.32%). Subgroup analysis showed that the impact of TBI severity, with the ES for moderate-severe TBI significantly higher than mild TBI (t (112) = 3.11, p = 0.002). Additionally, the component of visual attention was differentially affected by TBI (F (2, 120) = 10.25, p<0.0001); the ES for selective attention (ES = 1.13) and covert orientation of visual attention (ES = 1.14) were large, whilst for sustained attention, the ES was medium at 0.43. A subgroup analysis comparing outcome measures showed that reaction time (ES = 1.12) was significantly more affected compared to performance accuracy (ES = 0.43), F (1, 96) = 25.98, p<0.0001).

**Conclusion:**

Large and significant deficits in visual attention was found following TBI which can last for years after the initial injury. However, different components of visual attention were not affected to the same extent, with selective visual attention and orientation of visual attention most affected following TBI.

## Introduction

Traumatic brain injury (TBI) is a form of induced structural brain damage that is typically caused by an external force sufficient to disrupt normal brain function [1, 2]. It is estimated that 69 million people sustained a TBI annually worldwide, and this injury may result in death and disability depending on the severity and type of injury [3]. Although the improvement of medical care has increased, many individuals with TBI continue to experience a variety of disabilities that affect their daily living such as driving, reading, and quality of life [4].

TBI is frequently associated with a myriad of cognitive deficits, particularly affecting attention and memory processes [5, 6]. Commonly, cognitive processes have been assessed using visual tasks and stimuli because of the ease with which visual attention can be manipulated and measured. Additionally, established models have been proposed that have accounted for how visual attention operates detect and process of information [7, 8]. Visual attention typically refers to a set of different cognitive mechanisms/processes that allow the brain to selectively attend to and process information within the visual scene [9]. Particularly, visual attention aids in the selection of relevant information and inhibition of other irrelevant information.

Visual attention can be goal driven (endogenous/ top-down) in which there is a voluntary direction of attention to a specific location in the visual field (for review see: [10], or stimulus driven (exogenous/bottom-up) in which salient components of the stimulus (such as colour, contrast) attracts attention [11, 12]. The operation of visual attention has been traditionally described as a moveable spotlight or zoom lens which allows the brain to focus or attend to a small spatial area (space-based) within the visual field [13], or it can be selective for object features (object-based) that are not specific to a location [14, 15]. Visual attention, based on object features can be driven by stimulus properties such as colour and shape or reflect Gestalt principles such as object grouping and good continuation [16, 17].

While the definition of visual attention is still debatable, there is agreement regarding the multiple categories/components of attention which accounts the ways in which attention can be utilised/deployed to acquire information [18, 19]. For example, visual attention can be directed so that it is selective for certain information, divided across multiple objects, sustained over a period or oriented towards a specific location. Selective visual attention describes the ability to choose relevant visual information whilst simultaneously ignoring irrelevant information. This component of visual attention can be achieved by directing visual attention to specific object/objects in the environment [15]. Divided visual attention refers to the ability to attend to two or more stimuli at the same time [20]. Sustained visual attention relates to the ability to maintain attention to a specific visual stimulus/stimulus over time without fluctuation in performance [18, 19]. Finally, the orientation of visual attention refers to the ability to direct/allocate attention to specific spatial location in the visual field [21]. It is important to note that these different components of attention do not operate independently, but frequently in combination to acquire relevant information.

Evidence for deficits in attention following TBI has been observed for selective visual attention [21–24], divided visual attention [23], sustained visual attention and orientation of visual attention [25–28]. However, other studies have failed to find evidence for any deficits in visual attention [29–32]. Differences in outcomes between studies might be because of two main reasons. First, studies have assessed visual attention using patients with different TBI severities (e.g., mild, moderate, and severe), and have not considered the possible impact of TBI severity on visual attention. It is possible that deficits in visual attention are more pronounced in more severe cases of TBI, but that remains to be empirically and clearly established. Second, it is difficult to judge the impact of different methods and approaches employed to investigate visual attention processes in TBI patients on study outcomes. It is possible that methodological considerations such as task difficulty construct validity and stimulus design which is not equated across different studies may mean that some methods may be more effective in detecting visual attention deficits in TBI. For example, selective visual attention has been assessed using multiple tasks, including visual search, Useful field of view test (UFOV), and cancelation tasks [21–24, 30, 33], and the findings of these studies are mixed in regard to whether visual attention is affected following TBI. Indeed, our recent systematic review (see Walz et al.) has suggested that methodological approaches are a source of heterogeneity in studies that have examined attentional deficits following TBI.

Overall, it is possible that methodological differences, severity as well as factors such as post-injury period, age, and educational level between studies may be contributing factors in increase in the difficulty in understanding whether and how visual attention is affected by TBI [34, 35]. Additionally, the type of outcome measure may also be differentially affected by TBI. Previous studies have measured TBI patients on dependent variables such as performance accuracy, reaction time, completion time, and composite scores using an aggregate of numerous other measures (see [5, 28]). Importantly, it remains to be established whether these different outcome measures are affected by TBI to the same extent, and a source of variability between studies that employ different outcome measures.

Though previous studies have investigated visual attention following TBI, there is at present little consensus regarding the extent of this deficit and whether experimental factors such as the type of visual attention task, the component of visual attention investigated, and dependent variable(s) measured differentially affects study outcomes. In the present review, we conducted a qualitative and a meta-analysis to quantify the effect size relating visual attention deficits accompanying TBI, and how factors such as the component of visual attention, injury severity, outcome measure and post injury period contribute to the impact of TBI on visual attention deficit.

We conducted a comprehensive search for articles in which both patients with TBI and matched controls completed a visual attention task. Patients with TBI were defined and classified based on the Glasgow Coma Scale (GCS) and duration of post-traumatic amnesia (PTA). On GCS scale, the patient with the score between 13–15, loss of consciousness (LOC) less than 30 minutes, and PTA less than 24 hours is considered having mild TBI while a patient with the score between 9–12, LOC between 30 minutes and 24 hours, and PTA between 1 and 7 days is considered as moderate TBI. A patient is considered having severe TBI if the GCS score between 3–8 and LOC greater than 24 hours and PTA greater than 7 days [2, 36]. Studies that have assessed visual attention can be categorized into one of four components based on the theoretical distinctions in the visual attention literature. This categorization includes selective visual attention, divided visual attention, sustained visual attention, and covert orientation of visual attention [19, 20, 37].

## Methods

### Literature search and inclusion criteria

A comprehensive literature search was undertaken using electronic databases, including Medline, Scopus, PubMed, and Google Scholar databases. The search terms that were used to capture relevant articles include “visual attention”, “selective visual attention”, “divided visual attention”, “sustained visual attention”, “covert orientation of visual attention”, “attention allocation”, “traumatic brain injury”, “concussion” in various combinations. Search terms and strategy can be found in Table 1. Studies published between January 1980 to May 2021 that examined visual attention following TBI were included. A backward search was performed from the reference list of eligible studies to avoid any missing relevant article. This review was conducted according to PRISMA guidelines for reporting systematic review and meta-analysis [38] The study protocol was registered with PROSPERO (CRD42020199419).

**Table 1:** PICO principles and Medline search strategy employed in the present review and in accordance with the PRISMA statement.

**“PICO” principles**	
Population (P)	Adults over 18 years and over who have had a traumatic brain injury TBI (mild, moderate, or severe). Patients with TBI were defined and classified based on the score of a common scaling system known as Glasgow Coma Scale (GCS) and duration of post-traumatic amnesia (PTA). Studies that recruited participants under 18 years, participants who diagnosed with other neurologic conditions or non-human studies were excluded.
Intervention (I)	Human participants with TBI with no intervention versus healthy controls
Comparison (C)	Control participants with comparable age, gender, and IQ
Outcome (O)	Percentage accuracy and/or reaction time as measures of performance on the visual attention task
**Search strategy used in Medline database**	
1	Traumatic brain injury or TBI
2	Head injury
3	Concussion
4	Visual attention
5	Selective visual attention
6	Divided visual attention
7	Sustained visual attention
8	Covert orientation of visual attention
9	Attention allocation
10	1 or 2 or 3
11	4 or 5 or 6 or 7 or 8 or 9
12	10 and 11

**Fig 1:**
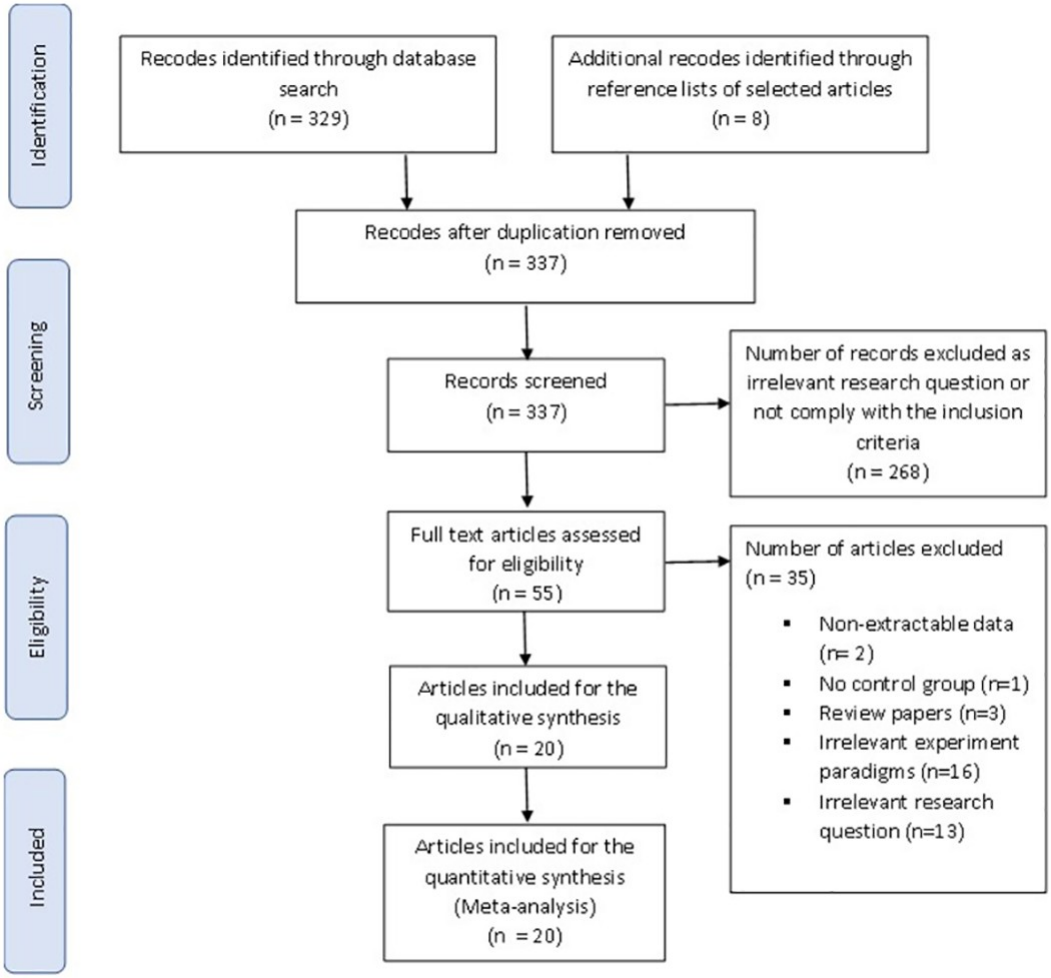
A schematic representation of the search and selection process for studies that were excluded and included in the present study.

Studies were included if they met the Population–Intervention–Comparison–Outcome (PICO) principle, as shown in Table 1 [39]. The meta-analysis was conducted using data only from published studies that fulfil the following criteria: (a) published in a peer-reviewed journal; (b) published in English language; (c) case-control study design; (d) if injury severity and post-injury period were provided; (e) studies that measured accuracy and/or reaction time as a performance measure of visual attention and (f) if study results were reported in an extractable format (means and standard deviations). Fig 1 shows the selection procedure for studies included for analysis. All studies included in this meta-analysis assessed visual attention using visual stimuli and those studies that used other sensory stimuli, such as auditory stimuli, were excluded. Case reports, case series, animal studies, systematic reviews, studies conducted on children and studies that used dual-task paradigm where participants perform two tasks simultaneously or used visual attention tasks that involve inhibitory control were also excluded. Studies adopting a dual-task paradigm was excluded in this review because they assess executive functions such as inhibition and switching which is known to be impaired following TBI [40–42], and typically both visual and auditory stimuli are used which does not meet the inclusion criteria of the review which included only studies that focussed only on vision.

Relevant articles were identified through the literature search by the first author (MA) and studies that met the inclusion criteria were extracted. The first author screened title and abstracts and consulted the last author (SKK) regarding the suitability of the articles for the study and for articles that involve complex visual attention tasks or articles that did not clearly report the component of visual attention that was investigated. The text was reviewed for eligible studies and data were extracted for further analysis.

### Data extraction and preparation

Data were extracted and collated into an electronic spreadsheet. The following information was extracted: name of the first author, year of publication, mean age of participants, mean and standard deviation (SD) of performance on the visual attention tests (accuracy or reaction time or both), TBI severity, post-injury period and sample sizes of cases and controls. The data was extracted using WebPlot Digitizer in studies that reported the mean and SD on graphs [43]. If the standard error of the mean (SEM) was reported in the graph, the SEM was converted to SD by multiplying SEM by the square root of the sample size. All studies included in the meta-analysis had complete datasets and reported descriptive statistics (i.e., means, SD, SEMs, and sample sizes) suitable for effect size calculations, and there were no missing data.

The Joanna Briggs Institute (JBI) appraisal for systematic reviews was used to assess the risk of bias for each individual study [44]. This tool is used for quality assessment, including methodological quality and possibility of bias in its design, conduct and analysis. The majority of included studies met the checklist requirements as shown in Table 2, and therefore, the risk of bias was low.

**Table 2:** The outcomes of an assessment of risk of bias of published studies included in the qualitative and quantitative analyses of the present review.

Author and year	Were the groups comparable other than the presence of disease in cases or the absence of disease in controls?	Were cases and controls matched appropriately?	Were the same criteria used for identification of cases and controls?	Was exposure measured in a standard, valid and reliable way?	Was exposure measured in the same way for cases and controls?	Were confounding factors identified?	Were strategies to deal with confounding factors stated?	Were outcomes assessed in a standard, valid and reliable way for cases and controls?	Was the exposure period of interest long enough to be meaningful?	Was appropriate statistical analysis used?
Robertson et al. (2017)	Yes	Yes	Yes	Yes	NA	Yes	No	Yes	Yes	Yes
Schmitter-Edgecombe et al. (1998)	Yes	Yes	Yes	Yes	NA	Yes	Yes	Yes	Yes	Yes
Malojcic et al. (2008)	Yes	Yes	Yes	Yes	NA	Yes	Yes	Yes	Yes	Yes
McIntire et al. (2006)	Yes	Yes	Yes	Yes	NA	Yes	Yes	Yes	Yes	Yes
Cremona-Meteyar et al. (1992)	Yes	Yes	Yes	Yes	NA	Yes	Yes	Yes	Yes	Yes
Bate et al. (2001)	Yes	Yes	Yes	Yes	NA	Yes	Yes	Yes	Yes	Yes
Donkelaar et al. (2005)	Yes	Yes	Yes	Yes	NA	Yes	Yes	Yes	Yes	Yes
Crenona-Meteyar et al. (1994)	Yes	Yes	Yes	Yes	NA	Yes	Yes	Yes	Yes	Yes
Hills et al. (1998)	Yes	Yes	Yes	Yes	NA	Yes	No	Yes	Yes	Yes
Pavlovskaya et al. (2007)	Yes	Yes	Yes	Yes	NA	Yes	Yes	Yes	Yes	Yes
Ponsford et al. (1992)	Yes	Yes	Yes	Yes	NA	Yes	Yes	Yes	Yes	Yes
Heinze et al. (1992)	Yes	Not clear	Not clear	Yes	NA	Yes	Yes	Yes	Yes	Yes
Halterman et al. (2006)	Yes	Yes	Yes	Yes	NA	Yes	Yes	Yes	Yes	Yes
Ziino et al. (2006)	Yes	Yes	Yes	Yes	NA	Yes	Yes	Yes	Yes	Yes
Slovarp et al. (2012)	Yes	Yes	Yes	Yes	NA	Yes	Yes	Yes	Yes	Yes
Wu et al. (2020)	Yes	Yes	Yes	Yes	NA	Yes	Yes	Yes	Yes	Yes
Stuss et al. (1989)	Yes	Yes	Yes	Yes	NA	Yes	Yes	Yes	Yes	Yes
Kim et al. (2009)	Yes	Yes	Yes	Yes	NA	Yes	Yes	Yes	Yes	Yes
Hill-Jarrett et al. (2015)	Yes	Yes	Yes	Yes	NA	Yes	Yes	Yes	Yes	Yes
Willmott et al. (2009)	Yes	Yes	Yes	Yes	NA	Yes	Yes	Yes	Yes	Yes

*Note*: Yes: Low risk of bias, No: high risk of bias; NA: Not applicable.

### Effect size calculations

Hedges’ g (Hedges, 1985) effect sizes were calculated for all visual attention performance results (accuracy or reaction time) by dividing the difference between the mean of the visual attention performance results of cases and controls by the pooled standard deviation (SD) [45]. The effect size 95% confidence interval (CI) was calculated following the method described by Hedges and Olkin [46]. Hedges’ g was used to estimate effect size (and not Cohen’s d) because a number of studies (8 out of 20) included in the present study had small or unequal sample sizes, which statistic takes into consideration. According to Cohen’s criteria, a small effect was defined if g<0.3, a moderate effect if g is 0.3–0.5, and a large effect g>0.5 [47]. A positive Hedges’ ES indicates the TBI group performed poorer than the control group, while a negative Hedges’ ES indicates TBI group performed better than the control group.

### Statistical analysis

Data were entered into Excel and analyses were performed using Excel workbooks provided by Meta-Essentials and Graph Pad Prism 8 Software [48]. The random effect model was used to determine the combined ES and standard error for eligible studies [49]. The same method was used to determine the combined effect size for the different components of visual attention (selective visual attention, sustained visual attention, and covert orientation of visual attention), TBI severity (mild and moderate–severe), and the type of outcome measures (accuracy and reaction time). To determine whether the combined ESs were statistically significant, one sample t-tests were conducted to determine if they were significantly different from zero. Unpaired t-test (parametric) were used to compare the combined ES for each severity and outcome measure. One-way ANOVA was used to compare the combined ESs of the different components of visual attention following TBI. Tukey’s post-hoc comparison tests were used for significant effects. Two-way ANOVAs were used to determine whether the combined ES for each outcome measure (accuracy and reaction time) is dependent on TBI severity (between mild and moderate-severe TBI) or component of visual attention (selective, sustained, and covert orientation of visual attention). Post-hoc analyses were conducted on significant effects using Sidak’s test for multiple comparisons.

The heterogeneity across studies was examined using the Q and I^2^ statistics [48]. The p-value was calculated using Q-statistics in which the null hypothesis of homogeneity is rejected if Q is significant. The degree of heterogeneity was estimated using the I^2^ in which values of 25%, 50% and 75% are considered to be low, medium and high heterogeneity respectively [50].

## Results

The literature search resulted in 329 potentially relevant articles. There were 55 abstracts that met the inclusion criteria. Fig 1 shows the selection process and search outcomes of the current meta-analysis [51]. A detailed review of the full text revealed a total of 20 articles that met the inclusion criteria [21, 22, 24–29, 31, 32, 52–61]. Studies that used visual attention tasks and involved inhibitory control, such as the Stroop and go-no-go task, dual attention and studies that report the performance as a composite score were excluded based on our exclusion criteria. One study was also excluded because the TBI group was significantly older than the control group [23].

### Qualitative analysis

Table 3 lists the 20 studies that were included in the systematic review and meta-analysis, along with a summary of participants, TBI severity, post-injury period, task used, outcome variables, and results.

**Table 3:** Participant summary, task characteristics, and results for the 20 reported articles.

First author	Year	TBI n	Control n	TBI severity	Post injury period (days)	Mean age (SD)		Visual attention task	Stimuli	Outcome variables	ES and significance
						TBI	Control				
Robertson et al.	2017	30	30	Moderate-Severe	12–89	30.4 (13.5)	29.8(12.8)	Visual search	Shapes	RT	1.17
Schmitter-Edgecombe et al.	1998	20	20	Moderate-Severe	>365	33.1 (10.1)	32.4(9.1)	Visual search	Letters	RT	1.66
Malojcic et al.	2008	37	63	Mild	80	31.3 (11.2)	36.0(12.1)	Continuous performance task	Letters	RT	0.95
McIntire et al.	2006	17	17	Mild	2	22 (4.2)	22(3.9)	RSVP task	Letters	Accuracy	0.17
Cremona-Meteyard et al.	1992	11	9	Moderate-Severe	365	29.3 (10.5)	30.1(9.4)	Posner cueing task	Red light flash	RT	1.38
Bate et al.	2001	35	35	Severe	843	28.9 (11.5)	30.2(10.3)	Posner cueing task	Red LEDs	RT	0.25
Donkelaar et al.	2005	20	20	Mild	2	21 (1.7)	21(1.8)	Attention network test (ANT)	Arrows	RT	0.56
Cremona-Meteyard et al.	1994	9	12	Mild	14	23 (4.2)	22.1(3.9)	Posner cueing task	Red light flash	RT	0.19
Hills et al.	1998	20	21	Severe	92	31.4 (9.8)	31.6(12.0)	Cancellation task	Letters and shapes	RT	1.08
Pavlovskaya et al.	2007	21	9	Severe	60–150	18–47 *	23–47*	Posner cueing task	Simple Figurers	RT	0.14
Ponsford et al.	1992	47	30	Severe	112	23.4 (7.4)	25.4(5.9)	Cancellation task	Letters	RT	1.01
										Accuracy	0.02
Heinze et al.	1992	11	20	Severe	>730	NA	NA	Visual search	Shapes	RT	2.49
										Accuracy	0.65
Halterman et al.	2006	20	20	Mild	2	21(1.74)	21.6(1.81)	Attention network test (ANT)	Arrows	RT	1.65
Ziino et al.	2006	46	46	Mild-Severe	240.3	35.3(13.1)	34.1(10.4)	Complex Selective Attention Task	Letters and Numbers	RT	0.98
Slovarp et al.	2012	9	9	Severe	1314	42.1 (12.2)	38.8(11.5)	digit cancellation test	Letters	RT	7.2
										Accuracy	0.24
Wu et al.	2020	45	50	Mild	7	43.8 (15.2)	43.9(15.2)	Cancellation task	Digits	RT	1.31
										ER	-0.37
Stuss et al.	1989	26	26	Mild-Severe	900	30.9 (11.9)	29.7 (12.4)	Simple/Multiple Choice Reaction Time Tests	Shapes	RT	0.62
Kim et al.	2009	17	15	Moderate	430	27.8 (9.8)	25.1(3.1)	Posner cueing task	Shapes	RT	4.89
										Accuracy	0.84
Hill-Jarrett et al.	2015	12	12	Moderate-to-severe	2100	28.7 (9.5)	25.1(9.8)	Attention network test (ANT)	Arrows	RT	0.86
										Accuracy	0.83
Willmott et al.	2009	40	40	Moderate to severe	47	26.3 (9.1)	28(9.8)	Selective Attention Task (SAT).	Letters and Numbers	RT	1.00

*Note*: n = number, ES = combined effect size, RT = reaction time, NA: No data available,

*age range.

#### Diagnostic criteria for TBI

The diagnostic criteria for classification of patients with TBI varied among included studies. The majority of studies have classified the severity of TBI based on either GCS scores, LOC, PTA, or a combination of these measures. Three studies used the American Academy of Neurology [31, 62]. This categorization system uses both the alteration of mental state and the period of loss of consciousness to classify TBI. One study used the Mild Traumatic Brain Injury Committee of the American Congress of Rehabilitation Medicine diagnoses criteria which is based on measures such as LOC, PTA, alteration in mental state, and the focal neurologic deficits [26, 56]. All included studies in the current meta-analysis classified injury severity as either mild, moderate, or severe TBI. Six studies investigated visual attention deficits in mild TBI, one study in moderate TBI, 5 studies in moderate-severe TBI, 6 studies in severe TBI, and 2 studies in different TBI severity including mild, moderate, and severe TBI.

#### Tasks used to assess visual attention

Several tasks were used to assess visual attention in patients with TBI in which each task differently targeted selective, sustained, and the covert orientation of visual attention. There were insufficient number of studies that investigated divided visual attention and most of the studies that assessed divided visual attention used dual task paradigm which did not meet our inclusion criteria.

Covert orientation of visual attention was assessed the most (n = 10), followed by selective visual attention (n = 7), while sustained visual attention was assessed in 4 different studies. The Posner cueing task was the most commonly used task to assess covert orientation of visual attention (n = 8) [25, 27, 28, 52, 54, 55, 62, 63]. Covert orientation of visual attention was also assessed by 2 studies using a modified Posner Cueing Task which was referred to as the visual non-search task [22, 29]. In this task, the subject was asked to identify a specific target among multiple distractors (e.g., an alphabet) and the location of the target is cued.

There were 3 studies that used visual search task to assess selective visual attention [22, 29, 53]. Selective visual attention was also assessed by 4 studies using a cancellation task [21, 24, 60, 64]. Sustained visual attention was assessed in 4 studies using continuous performance task, digit cancellation test, or rapid serial visual presentation (RSVP) task [31, 56, 58, 59].

#### Participants

Data from a total of 1049 participants from 20 studies contributed to the meta-analysis. These studies included 519 patients with TBI and 530 control participants. All studies included in the current meta-analysis matched both groups based on moderator variables, such as age, gender, and education level. There were 3 studies who matched participants based on the IQ scores in addition to age, gender, and education level [27, 28, 52]. One study matched participants based on their parent’s occupational status [22]. A large number of the studies recruited TBI patients with different causes, including falls, assaults, and/or motor vehicle accident [22, 27, 29, 54, 58–61, 64]. Four studies recruited patients with sports related TBI [31, 26, 52]. Six studies that did not report the cause of TBI injury [21, 24, 25, 28, 55, 61].

#### Publication bias

**Fig 2:**
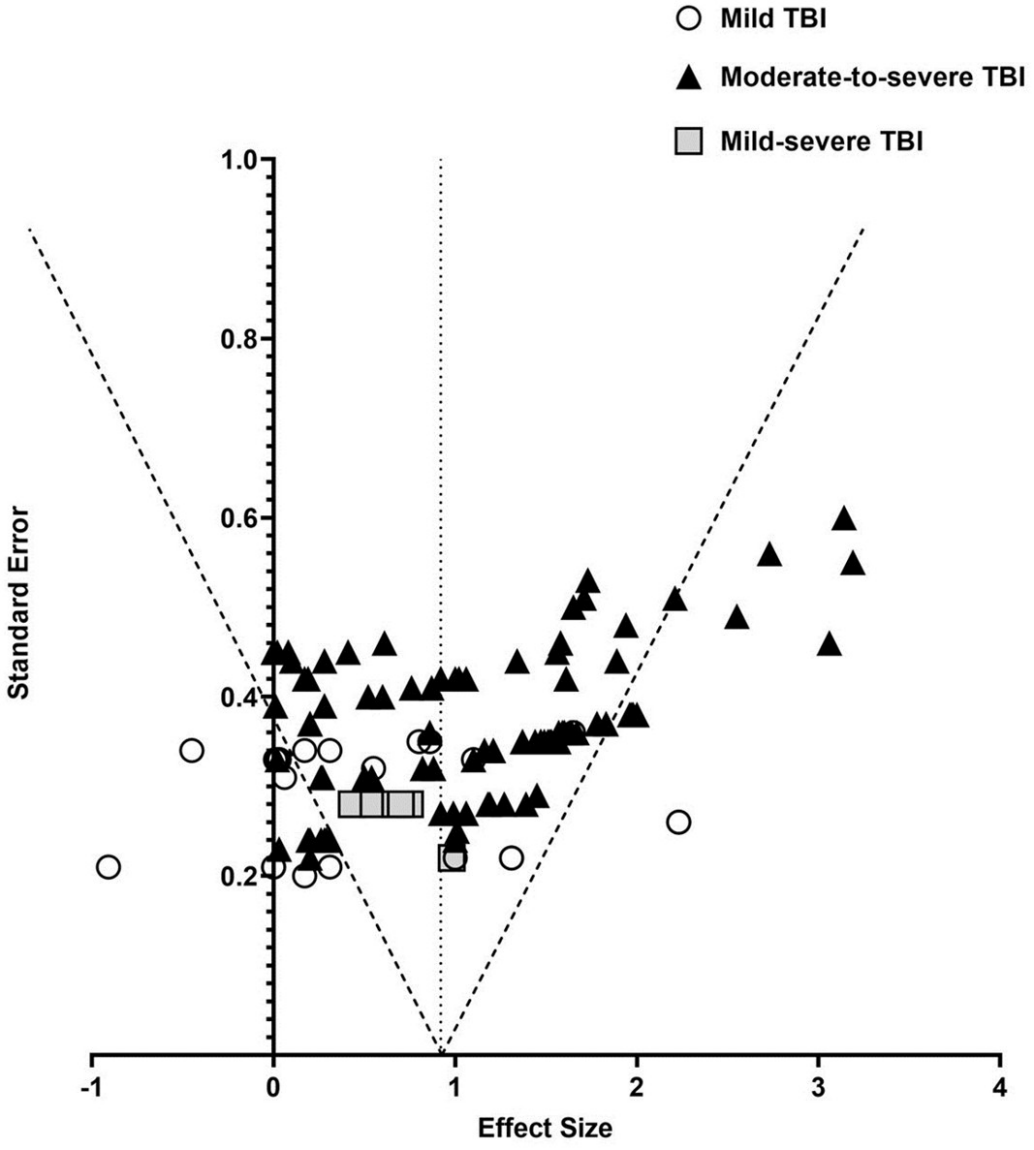
Funnel plot for publication bias. Funnel plot analysis, indicating potential publication bias. The different symbols represent the severity subgroups which may account for this bias.

Publication bias was assessed by a visual inspection of a funnel plots, and ESs that were beyond 2 SD from the combined ES were excluded. A large number of studies did fall outside the 95% error intervals, as shown in Fig 2, which indicates that there is a bias in the studies included in the meta-analysis. A regression asymmetry test showed that there was significant asymmetry (Egger’s regression test, p < 0.0001). We attribute the significant publication bias to the fact that there were different TBI severity among the included studies as shown in Fig 2.

### Quantitative analysis (Meta-Analysis)

All twenty studies were included in the meta-analysis. Effect size was calculated for each performance outcome measure such as performance accuracy and reaction time and combined ESs were determined for severity (mild, moderate-severe, and severe TBI), outcome measure (performance accuracy and reaction time) and component of visual attention (selective, sustained, and covert orientation of visual attention; see Introduction for definitions). Most studies contributed more than one ES measure, which may represent different task conditions and different time points. For longitudinal studies in which visual attention was assessed more than one occasion to measure recovery, only results from the first visit was used, because we were interested in the initial deficit and not any change due to practice or recovery.

#### Visual attention following TBI

**Fig 3:**
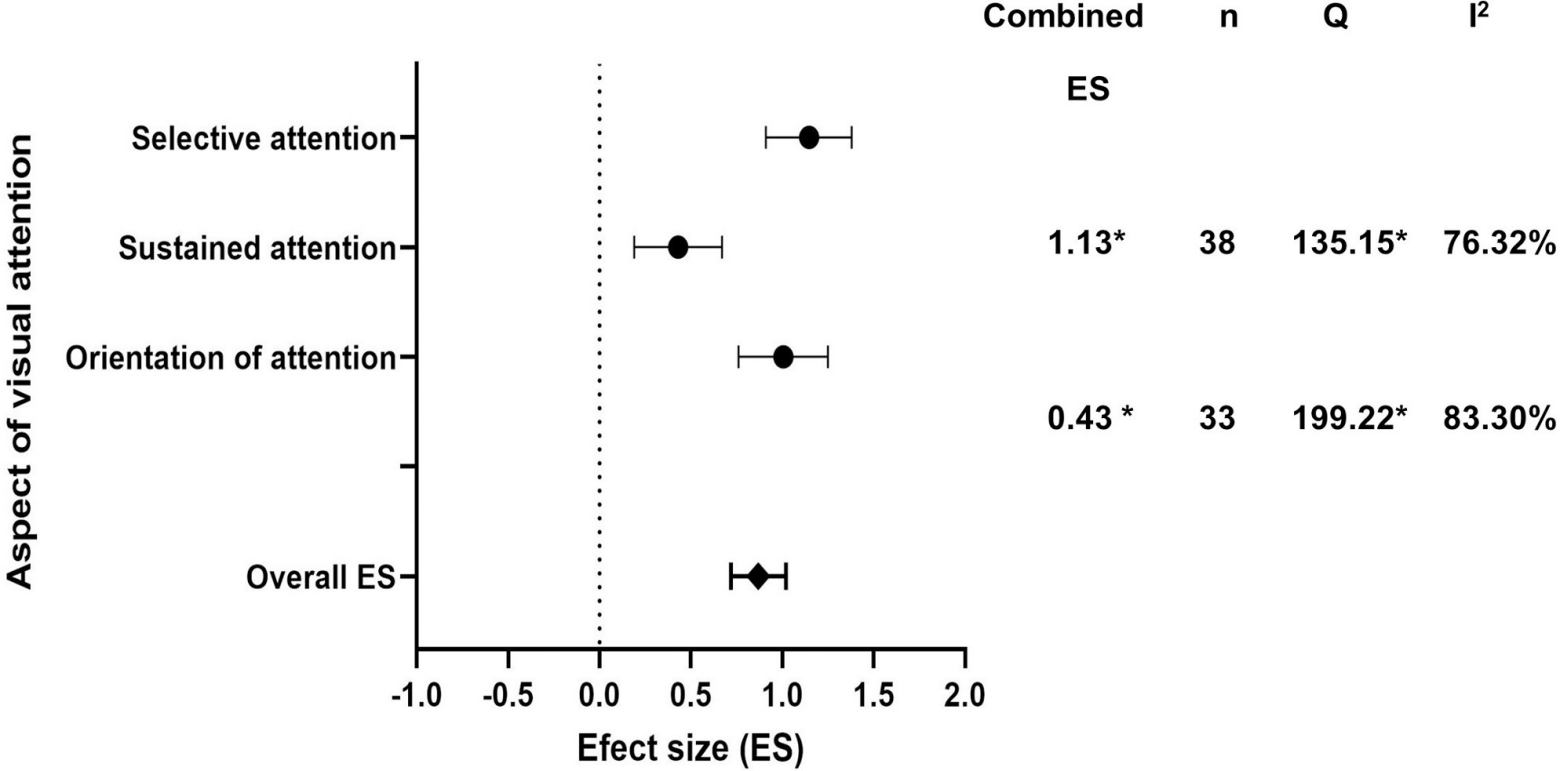
Forest plot showing overall and combined effect size. Forest plot of combined ES, 95% CI, number visual attention tasks (n), heterogeneity (Q) and the magnitude of heterogeneity (I2) of overall, selective visual attention, sustained visual attention, and the orientation of visual attention. Note: *p <0.01.

Table 4 reports the results of the overall ES and the combined ESs for different components of visual attention, TBI severity, and outcome measures, and these values are plotted in Fig 3. The 20 studies result in a total of 123 ESs represent different components of visual attention and conditions. The overall impact of TBI on visual attention was significant and was large (ES = 0.92, SE = 0.09, p<0.0001), but with significant heterogeneity (Q = 614.83, p < 0.0001, I^2^ = 80.32%). Also refer S1 Fig for forest plots of individual effect sizes of all components of visual attention.

**Table 4:** Key results from the meta-analysis classified in terms of TBI severity, the component of visual attention and the type of outcome measure.

		n	Combined ES	95% CI		Q	I^2^
				Lower	Upper		
**Component of visual attention**	Selective visual attention	38	1.13*	0.90	1.35	154.01*	75.98%
	Sustained visual attention	33	0.43***	0.19	0.67	199.22*	83.94%
	Covert orientation of visual attention	52	1.14*	0.91	1.37	225.54*	77.39%
**TBI severity**	Mild	25	0.59**	0.31	0.88	157.82*	84.79%
	Moderate—severe	89	1.12*	0.95	1.28	367.85*	76.08%
**Task outcome measures**	Accuracy	34	0.37**	0.23	0.70	91.60*	63.98%
	Reaction time	89	1.12*	0.53	1.47	351.07*	79.78%
**Overall**		123	0.92*	0.79	1.06	614.83*	80.32%

*Note*: n = number of effect sizes, ES = effect size,

*Significant at 0.001 level,

** Significant at 0.01 level,

*** Significant at 0.05 probability.

A one-way ANOVA revealed a significant difference between different components of visual attention (F (2, 120) = 10.25, p<0.0001). Tukey’s multiple comparisons test revealed that the estimated combined ES for selective visual attention significantly different from and larger than the ES for sustained visual attention [mean difference (MD) = -0.70, p = 0.0006] but not significantly different from covert orientation visual attention [mean difference (MD) = 0.01, p = 0.97]. The estimated combined ES for covert orientation visual attention was also significantly different from and larger than the ES for sustained visual [mean difference (MD) = -0.71, p = 0002].

#### Subgroup analyses: Effect of severity and outcome measure

We examined the combined ESs based on TBI severity. Note that there was insufficient data to further categorise studies on the components of visual attention. Instead, we simply divided TBI severity into mild and moderate-severe as per conventional reporting. The combined ES for mild TBI was significant and moderate (ES = 0.59, SE = 0.15 p < 0.001) but with large heterogeneity (Q = 157.82, p < 0.0001, I^2^ = 84.79%). For moderate-severe TBI, the combined ES was significant with large (ES = 1.12, SE = 0.08, p < 0.001) and with large heterogeneity (Q = 367.85, p < 0.0001, I^2^ = 76.08%). An unpaired t-test was used to compare the ES for mild and moderate-severe TBI groups. This analysis showed that both groups were significantly different, and the moderate-severe group had a larger effect size than the mild TBI group (t (112) = 3.11, p = 0.002), see Table 4.

The combined ES for each outcome measure (accuracy and reaction time) is shown in Table 4. The combined ES for accuracy was significant, with a medium ES of 0.37, (SE = 0.12, p < 0.001). There was significant and moderate heterogeneity among the studies that report the accuracy of performance (Q = 91.60, p<0.001, I^2^ = 63.98%). The combined ES for reaction time was significant and large with an ES of 1.12, (SE = 0.08, p<0.001) with a large heterogeneity (Q = 351.07, p < 0.0001, I^2^ = 79.78%). An unpaired t-test confirmed that reaction times were significantly more affected by TBI than accuracy (t = 5.026, df = 121, p<0.0001).

We additionally performed a sensitivity analysis to examine the robustness of the results for each outcome measure (accuracy and reaction time). This aids in determining whether the combined ES and its p-value for each outcome measure by systematically excluding one study in turn, to rule out the possibility that the effect is driven by one study. The results of this sensitivity analysis are shown in Tables 5 and 6 for accuracy and reaction time outcome measures. Importantly, this analysis showed that the ES for both outcome measures remained moderate and significantly different from 0.

**Table 5:** Outcomes of a sensitivity analysis of studies in which the combined effect size for task accuracy was estimated.

First author	Combined ES	95% CI		p
		Lower	Upper	
McIntire et al. (2006)	0.50	0.20	0.79	P < 0.0001
Ponsford et al. (1992)	0.40	0.17	0.62	P < 0.0001
Heinze et al. (1992)	0.35	0.11	0.13	P = 0.001
Slovarp et al. (2012)	0.43	0.12	0.69	P < 0.0001
Wu et al. (2020)	0.39	0.17	0.62	P < 0.0001
Kim et al. (2009)	0.36	0.14	0.58	P < 0.0001
Hill-Jarrett et al. (2005)	0.30	0.08	0.50	P = 0.003

**Table 6:** The outcomes of a sensitivity analysis of studies used to estimate the combined effect size for reaction time.

First author	Combined ES	95% CI		p
		Lower	Upper	
Robertson et al. (2017)	1.11	0.94	1.28	P < 0.0001
Schmitter-Edgecombe et al. (1998)	0.89	0.71	1.07	P < 0.0001
Malojcic et al. (2008)	1.13	0.98	1.28	P < 0.0001
Cremona-Meteyard et al. (1992)	1.10	0.95	1.26	P < 0.0001
Bate et al. (2001)	1.20	1.04	1.35	P < 0.0001
Donkelaar et al (2005)	1.14	0.98	1.29	P < 0.0001
Cremona-Meteyard et al. (1994)	1.16	1.00	1.31	P < 0.0001
Hills et al. (1998)	1.12	0.96	1.28	P < 0.0001
Pavlovskaya et al. (2007)	1.14	0.98	1.29	P < 0.0001
Ponsford et al. (1992)	1.12	0.96	1.27	P < 0.0001
Heinze et al. (1992)	1.07	0.92	1.22	P < 0.0001
Halterman et al. (2006)	1.11	0.95	1.26	P < 0.0001
Ziino et al. (2006)	1.12	0.96	1.27	P < 0.0001
Wu et al. (2020)	1.14	0.97	1.30	P < 0.0001
Stuss et al. (1989)	1.15	0.99	1.31	P < 0.0001
Hill-Jarrett et al. (2015)	1.13	0.97	1.29	P < 0.0001
Willmott et al. (2009)	1.12	0.96	1.27	P < 0.0001

**Fig 4:**
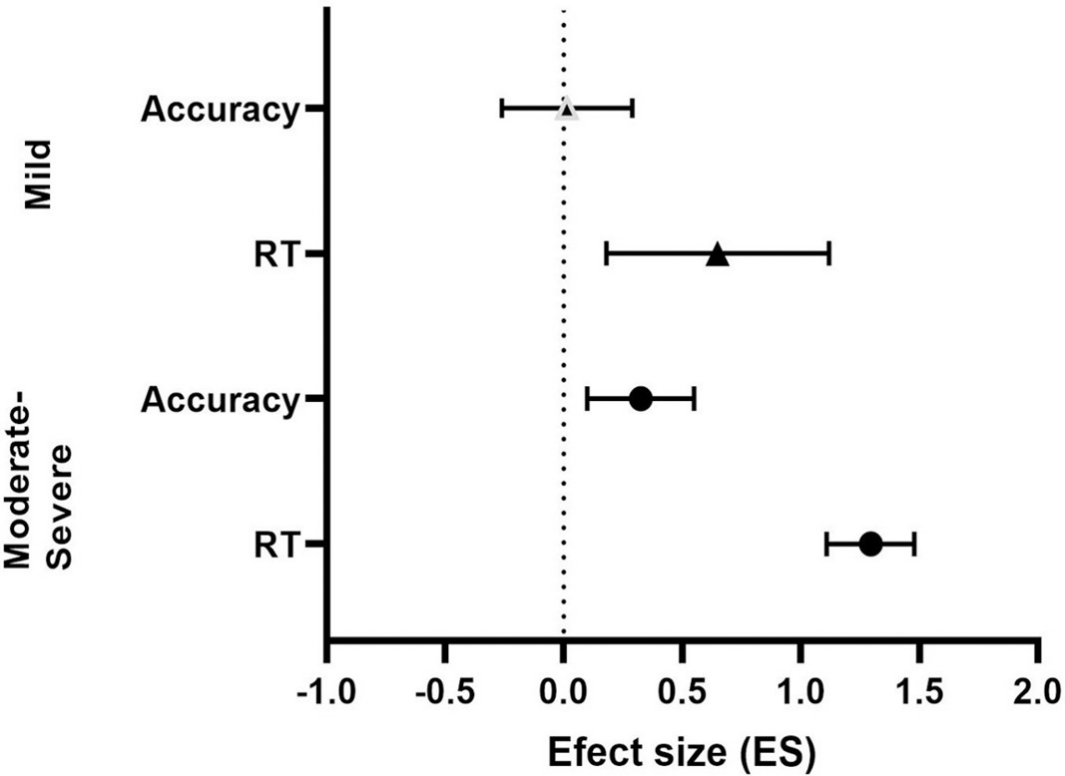
A forest plot showing subgroup analysis based on TBI severity (mild and moderate-severe) for accuracy and reaction combined effect-sizes.

Further analysis was conducted to determine whether the combined ES for each outcome measure (accuracy and reaction time) was dependent on TBI severity (between mild and moderate-severe TBI) which is shown in Fig 4. A 2-way ANOVA revealed a significant main effect for TBI severity (F (1, 112) = 16.75, p<0.001) and a main effect for outcome measures (F (1, 92) = 11.45, p = 0.001). No significant interaction was observed (F (1, 112) = 0.1889, p = 0.66). Sidak’s multiple comparisons post-hoc test showed that the combined ES for reaction time was significantly more impacted compared to accuracy in both mild TBI (MD = -0.64, p = 0.043) and moderate-severe TBI (MD = -0.50, p = 0.006).

**Fig 5:**
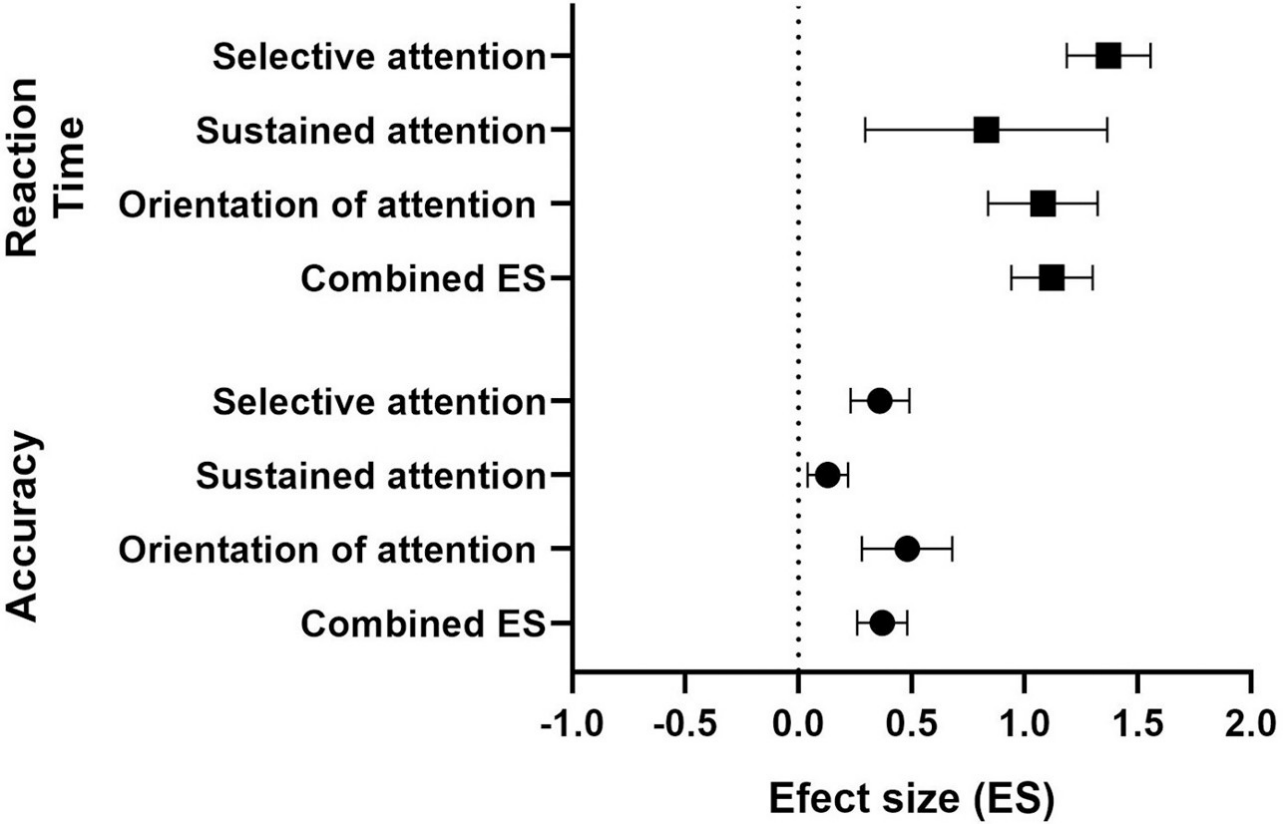
Forest plot showing subgroup analysis of different the components of visual attention. Forest plot of combined effect size, 95%CI, for accuracy and reaction time for selective visual attention, sustained visual attention, and the orientation of visual attention.

A subgroup analysis was also conducted to determine whether the combined ES for accuracy and reaction time outcome measures was dependent on the component of visual attention, and these combined ESs are shown in Fig 5. A 2-way ANOVA revealed a significant main effect of outcome measure (F (1, 116) = 34.19, p<0.0001), and no significant effect regarding the component of visual attention (F (2, 116) = 3.033, p = 0.052) nor a significant interaction effect (F (2, 116) = 0.86, p = 0.43). Sidak’s multiple comparisons post-hoc test showed that the combined ES for reaction time was significantly more impacted compared to accuracy across all components of visual attention (selective visual attention: MD = -1.01, p = 0.0002; sustained visual attention: MD = -0.70, p = 0.009; covert orientation of visual attention: MD = -0.60, p = 0.017).

#### Influence of post-injury period duration

**Fig 6:**
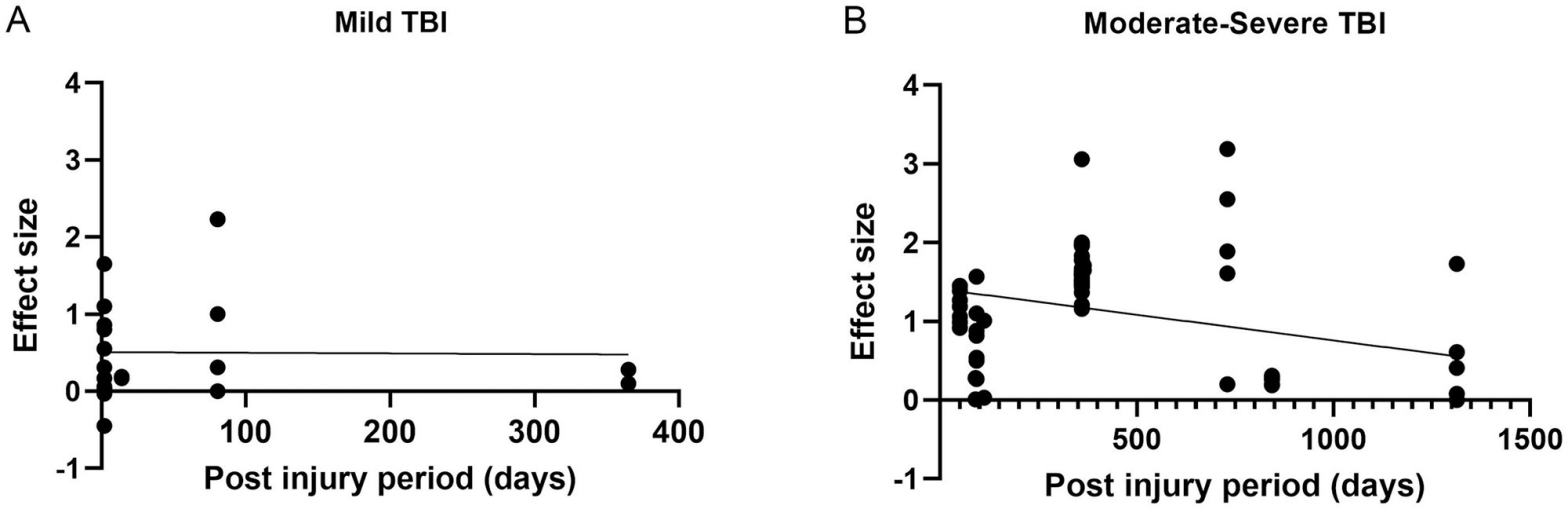
Meta-regression analyses between effect sizes and post injury period in the two main severity subgroups. Fig 6A shows effect size estimates for studies of mild TBI and Fig 6B shows that of moderate-to-severe TBI. Each figure includes the line of best fit for each group.

A meta-regression analysis was conducted to assess the effect of post-injury period on the performance in visual attention for different TBI severity (i.e., patients with mild or moderate-severe TBI). There was insufficient data to consider this analysis for different components of visual attention. The results of the meta-regression analyses are presented in Fig 6. A total of 114 effect sizes estimated from the 20 studies were included for the regression analysis and were grouped based on the TBI severity. In this analysis, two studies [59, 61] were excluded as they included groups of patients with mixed TBI severities.

For mild TBI, the relationship between effect sizes and post-injury period was not significant as shown in Fig 6A (R^2^ = 0.00015, p = 0.96). In addition, the regression line intercepts the X-axis at 5979 days or approximately 16 years, suggesting that the recovery from visual attention deficits might not occur at least within the first three months following the initial injury and remains unchanged for longer periods. In contrast, in moderate-severe TBI, the relationship between effect sizes and post-injury period was statistically significant, (R^2^ = 0.1245, p = 0.003) as shown on Fig 6B, and the regression line intercepts the X-axis at 2167 days or approximately 6 years indicating improvements in visual attention over time.

## Discussion

In the current review, a qualitative and quantitative analysis was undertaken to investigate collectively whether and how visual attention is affected by TBI. In particular, we investigated whether factors such as the component of visual attention, TBI severity, and visual attention outcome measures (particularly accuracy and reaction times) differentially affect study outcomes. The meta-analysis comprised of 123 effect sizes from 20 studies that met the study inclusion criteria. This analysis showed that visual attention can be greatly impacted by TBI, as indicated by a large combined effect size (ES = 0.92), but there was significant heterogeneity (Q = 614.83, p < 0.0001, I^2^ = 80.32%), which perhaps suggests the role of a number of factors contributing to the variability between studies.

The finding of an overall large effect indicates that visual attention can be greatly impaired in patients with TBI. Our findings agree with and is supported by previous meta-analyses and systematic reviews that assessed attention following TBI [5, 10, 65, 66]. However, these studies included studies that assess auditory attention or attention tasks that involve both visual and auditory stimuli, which were excluded from our analysis. Our findings are unique as they show adverse effects of TBI on visual attention only, and raise the possibility of using visual attention tasks in the characterisation of TBI in research and clinical practices.

Our review reports (to our knowledge) for the first time how different components of visual attention are affected following TBI. The combined effect size for tasks that target specific visual attention, including selective, sustained and the covert orientation of visual attention, were significant and different from each other (selective attention: ES = 1.13; covert orientation of visual attention; ES = 1.14; sustained attention; ES = 0.43). The current work findings suggest TBI leads to large deficits in selective visual attention in which both visual search and cancelation tasks were used to distinguish performance between TBI patients from normal controls. TBI also produced clear deficits in the ability to covertly orientate visual attention to a specific spatial location. Particularly, TBI patients may not benefit from valid spatial cueing in a Posner cueing task, which is consistent with our recent work that showed a clear deficits in the visuospatial attention [10]. It is apparent that both selective visual attention and to the covert orientation of visual attention were similarly affected and this may be due to the fact that both tasks require the allocation of visual to either a specific visual stimulus/stimulus or to a specific location, see [67]. In contrast, the magnitude of deficit for sustained visual attention was less as compared to the other components of visual attention, though this component was nevertheless significant and moderate.

In our review, we performed a subgroup analysis to determine whether task accuracy and reaction time outcome measures were equally affected following TBI and for different components of visual attention. In general, we found that the combined effect size for reaction time (ES = 1.12) was significantly larger than the combined effect size for accuracy (ES = 0.37), and subgroup analyses showed that this trend was the same regardless of the component of visual attention (see Results). This reduction in the processing speed is a common report in TBI studies, and may be a primary deficit of brain injury [5]. Our results suggest that reaction time measures might be more sensitive in detecting subtle change in visual attention compared to accuracy following TBI.

Subgroup analysis additionally showed that TBI severity also affected visual attention. The combined effect size for mild TBI (ES = 0.59) was significantly smaller than moderate-severe TBI (ES = 1.12). TBI patients included in the present review were classified using current systems, such as GCS score, and highlight their usefulness to assess the initial injury. Their utility (and in relation to attention) may be due to the fact that the current classification systems typically involve a behavioural examination of consciousness and awareness, which requires attention. However, future research is needed to examine further whether the initial GCS score is associated with performance on different visual attention tasks.

We were unable to subgroup different component of visual attention based on the TBI severity because of insufficient data. When accuracy and reaction time outcome measures were examined separately for mild TBI, interestingly, we find a clear deficit in reaction time (ES = 0.65) but no significant difference in task accuracy (ES = 0.01). This suggest mild TBI adversely affects processing speed but not necessarily the ability to accurately perform the task. In contrast, moderate-severe TBI led to deficits in both outcome measures, but the magnitude was greater for reaction time (ES = 1.30) than task accuracy (ES = 0.30). These findings reiterate that processing speed is more susceptible to brain injury, and task accuracy is affected when the severity of injury becomes greater.

Our qualitative analysis showed that different visual stimuli were used across the studies that met our inclusion criteria. It is important to consider the type of visual stimuli because previous studies have reported differences in low and high level visual processing in TBI patients [68–71]. It is important to note that all included studies in our meta-analysis used visual stimuli that require low-level processing and typically required the detection letters and shapes, and not higher level visual-processing such as global form or motion detection. Thus, it is unlikely that differences in study outcomes can be attributed to differences in the stage at which visual information is processed.

Our meta-regression analysis results showed that deficits in visual attention from TBI did not improve over time and may last for up to 16 years in individuals with mild TBI (see Fig 6A). Importantly, this result indicated that, whilst it has been reported that mild TBI patients typically recover 1 to 3 months post injury [72, 73], deficits in visual attention are likely to be more enduring. In contrast, significant improvement was observed in visual attention for moderate-severe TBI patients. This might be attributed to the fact that given the severity of injury patients with moderate-severe TBI may receive early intervention and more medical attention such as comprehensive neuro rehabilitation compared to patients with mild TBI [74–76]. Neuro rehabilitation programs have been shown to improve attention and other cognitive function and future studies may wish to compare visual attention performance in TBI patients who have or have not received neuro rehabilitation treatments or any other therapies (see [77]), with the possibility that such treatments may improve visual attention deficits. In the current review, we were unable to investigate whether neuro rehabilitation improves visual attention since the majority of the studies included in our meta-analysis did not report, or there was insufficient information regarding the TBI patients received treatment.

### Limitations

The meta analysis reported in the present study had a number of limitations. A significant high heterogeneity between studies was observed which cannot be immediately explained (I^2^>75%). Medium to high heterogeneity was still observed after subgroup analyses that considered the component of visual attention, TBI severity and outcome measures as possible sources of variation. Additional sources of heterogeneity might be the diagnostic criteria for TBI which was not consistent across all studies. For example, some studies used only one or multiple criteria including GCS, LOC, PTA, or other diagnostic tests from the American Academy of Neurology or Mild Traumatic Brain Injury Committee of the American Congress of Rehabilitation Medicine. Importantly, these criteria may assess and emphasise different functional categories in the diagnosis of TBI, and which may not reflect those critical to visual attention. In addition, TBI aetiology was not reported in all studies, which might be another major source of heterogeneity. Another source of variability is related to differing stimulus conditions. For example, some studies measured performance across multiple stimulus conditions or time points which is likely to introduce systematic bias.

## Supporting information

S1 Fig Individual effect sizes. Individual effect sizes and combined effect sizes for each component of visual attention (selective A, sustained B, orientation of attention C.
(PDF)

S1 Table PRISM check list utilized for the current systematic review and meta-analysis. (PDF)

S1 Data Used for the meta-analysis. (XLSX)

## References

[1] Corrigan J.D., Selassie A.W., and Orman J.A.L. The epidemiology of traumatic brain injury. *The Journal of head trauma rehabilitation*. (2010). 25(2): 72–80. doi: 10.1097/HTR.0b013e3181ccc8b4 20234226

[2] Statements Q. VA/DoD clinical practice guideline for management of concussion/mild traumatic brain injury. *Rehabil Res Dev*. (2009). 46(6): 1–60. 20108447

[3] Dewan M.C., et al. Estimating the global incidence of traumatic brain injury. *Journal of neurosurgery*. (2018). 130(4): 1080–1097. doi: 10.3171/2017.10.JNS17352 29701556

[4] Zaloshnja E., et al. Prevalence of long-term disability from traumatic brain injury in the civilian population of the United States, 2005. *The Journal of head trauma rehabilitation*. (2008). 23(6): 394–400. doi: 10.1097/01.HTR.0000341435.52004.ac 19033832

[5] Mathias J.L. and Wheaton P. Changes in Attention and Information-Processing Speed Following Severe Traumatic Brain Injury. *Neuropsychology*. (2007). 21(2): 212–223. https://psycnet.apa.org/doi/10.1037/0894-4105.21.2.212. 17402821 10.1037/0894-4105.21.2.212

[6] Rabinowitz A.R. and Levin H.S. Cognitive sequelae of traumatic brain injury. *The Psychiatric Clinics of North America*. (2014). 37(1): 1–11. doi: 10.1016/j.psc.2013.11.004 24529420 PMC3927143

[7] Posner M.I., Snyder C.R., and Davidson B. Attention and the detection of signals. *Journal of experimental psychology*. (1980). 109(2): 160. https://psycnet.apa.org/doi/10.1037/0096-3445.109.2.160. 7381367

[8] Treisman A.M. Strategies and models of selective attention. *Psychological review*. (1969). 76(3): 282. https://psycnet.apa.org/doi/10.1037/h0027242. 4893203 10.1037/h0027242

[9] Evans K.K., et al. Visual attention. *Wiley Interdisciplinary Reviews*: *Cognitive Science*. (2011). 2(5): 503–514. doi: 10.1002/wcs.127 26302302

[10] Walz J.A., et al. Visuospatial attention allocation as an indicator of cognitive deficit in traumatic brain injury: a systematic review and meta-analysis. *Frontiers in human neuroscience*. (2021). 402. doi: 10.3389/fnhum.2021.675376 34354575 PMC8329082

[11] Connor C.E., Egeth H.E., and Yantis S. Visual attention: bottom-up versus top-down. *Current biology*. (2004). 14(19): R850–R852. doi: 10.1016/j.cub.2004.09.041 15458666

[12] Yantis S. and Egeth H.E. On the distinction between visual salience and stimulus-driven attentional capture. *Journal of Experimental Psychology*: *Human Perception and Performance*. (1999). 25(3): 661. https://psycnet.apa.org/doi/10.1037/0096-1523.25.3.661. 10385983 10.1037//0096-1523.25.3.661

[13] Eriksen C.W. and Yeh Y.-y. Allocation of attention in the visual field. *Journal of Experimental Psychology*: *Human Perception and Performance*. (1985). 11(5): 583. doi: 10.1037//0096-1523.11.5.583 2932532

[14] Duncan J. Selective attention and the organization of visual information. *Journal of Experimental Psychology*: *General*. (1984). 113(4): 501. https://psycnet.apa.org/doi/10.1037/0096-3445.113.4.501. 6240521 10.1037//0096-3445.113.4.501

[15] Yantis S. and Johnston J.C. On the locus of visual selection: evidence from focused attention tasks. *Journal of experimental psychology*: *Human perception and performance*. (1990). 16(1): 135. https://psycnet.apa.org/doi/10.1037/0096-1523.16.1.135. 2137515 10.1037//0096-1523.16.1.135

[16] Vecera S.P. and Farah M.J. Does visual attention select objects or locations? *Journal of Experimental Psychology*: *General*. (1994). 123(2): 146. doi: 10.1037//0096-3445.123.2.146 8014610

[17] Baylis G.C. and Driver J. Visual parsing and response competition: The effect of grouping factors. *Perception & psychophysics*. (1992). 51(2): 145–162. doi: 10.3758/bf03212239 1549433

[18] Robertson I.H., et al. The structure of normal human attention: The Test of Everyday Attention. *Journal of the International Neuropsychological Society*. (1996). 2(6): 525–534. doi: 10.1017/s1355617700001697 9375156

[19] Posner M.I. and Petersen S.E. The attention system of the human brain. *Annual Review of Neuroscience*. (1990). 13(1): 25–42. doi: 10.1146/annurev.ne.13.030190.000325 2183676

[20] Schneider W. and Shiffrin R.M. Controlled and automatic human information processing: I. Detection, search, and attention. *Psychological review*. (1977). 84(1): 1. https://psycnet.apa.org/doi/10.1037/0033-295X.84.2.127.

[21] Hills E.C. and Geldmacher D.S. The effect of character and array type on visual spatial search quality following traumatic brain injury. *Brain Injury*. (1998). 12(1): 69–76. doi: 10.1080/026990598122872 9483339

[22] Schmitter-Edgecombe M., Kibby, and Michelle K. Visual selective attention after severe closed head injury. *Journal of the International Neuropsychological Society*. (1998). 4(2): 144–159. doi: 10.1017/s1355617798001441 9529824

[23] Fisk G.D., et al. Useful Field of View After Traumatic Brain Injury. *The journal of head trauma rehabilitation*. (2002). 17(1): 16–25. doi: 10.1097/00001199-200202000-00004 11860326

[24] Geldmacher D.S. and Hills E.C. Effect of stimulus number, target-to-distractor ratio, and motor speed on visual spatial search quality following traumatic brain injury. *Brain Injury*. (1997). 11(1): 59–66. doi: 10.1080/026990597123818 9012552

[25] Pavlovskaya M., et al. Hemispheric visual atentional imbalance in patients with traumatic brain injury. *Brain and cognition*. (2007). 64(1): 21–29. doi: 10.1016/j.bandc.2006.10.003 17182160

[26] van Donkelaar P., et al. Attentional deficits in concussion. *Brain Injury*. (2005). 19(12): 1031–1039. doi: 10.1080/02699050500110363 16263646

[27] Cremona-Meteyard S. L., et al. Covert orientation of visual attention after closed head injury. *Neuropsychologia*. (1992). 30(2): 123–132. https://psycnet.apa.org/doi/10.1016/0028-3932(92)90022-E. 1560891 10.1016/0028-3932(92)90022-e

[28] Bate A.J., Mathias J.L., and Crawford J.R. The Covert Orienting of Visual Attention Following Severe Traumatic Brain Injury. *Journal of Clinical and Experimental Neuropsychology*. (2001). 23(3): 386–398. doi: 10.1076/jcen.23.3.386.1190 11404815

[29] Robertson K. and Schmitter-Edgecombe M. Focused and divided attention abilities in the acute phase of recovery from moderate to severe traumatic brain injury. *Brain Injury*. (2017). 31(8): 1069–1076. doi: 10.1080/02699052.2017.1296192 28481625 PMC6174004

[30] Schneider J.J. and Gouvier W.D. Utility of the UFOV Test With Mild Traumatic Brain Injury. *Applied Neuropsychology*. (2005). 12(3): 138–142. doi: 10.1207/S15324826AN1203_3 16131340

[31] McIntire A., et al. The influence of mild traumatic brain injury on the temporal distribution of attention. *Experimental Brain Research*. (2006). 174(2): 361–366. doi: 10.1007/s00221-006-0469-8 16676168

[32] Wu Y., Zhang Y., and Wang Y. Multiple component analysis of attention early after complicated mild traumatic brain injury: a pilot study. J.J.o.r.m (2020). doi: 10.2340/16501977-2673 32266412

[33] Adelson E.H. and Movshon J.A. Phenomenal coherence of moving visual patterns. *Nature*. (1982). 300(5892): 523. doi: 10.1038/300523a0 7144903

[34] McCalley L., Bouwhuis D., and Juola J.F. Age changes in the distribution of visual attention. *The Journals of Gerontology Series B*: *Psychological Sciences Social Sciences* (1995). 50(6): P316–P331. doi: 10.1093/geronb/50b.6.p316 7583811

[35] Wiegand I., et al. Neural correlates of age-related decline and compensation in visual attention capacity. *Neurobiology of Aging*. (2014). 35(9): 2161–2173. doi: 10.1016/j.neurobiolaging.2014.02.023 24684790

[36] Teasdale G. and Jennett B. Assessment of coma and impaired consciousness: a practical scale. *The Lancet*. (1974). 304(7872): 81–84. doi: 10.1016/s0140-6736(74)91639-0 4136544

[37] Yantis S. Multielement visual tracking: Attention and perceptual organization. *Cognitive psychology*. (1992). 24(3): 295–340. doi: 10.1016/0010-0285(92)90010-y 1516359

[38] Page M.J., et al. The PRISMA 2020 statement: an updated guideline for reporting systematic reviews. *Bmj*. (2021). 372. doi: 10.1136/bmj.n71 33782057 PMC8005924

[39] Wright R.W., et al. How to write a systematic review. (2007). 455: 23–29. 10.1097/BLO.0b013e31802c9098 17279036

[40] Bull R., Phillips L.H., and Conway C.A. The role of control functions in mentalizing: Dual-task studies of theory of mind and executive function. *Cognition*. (2008). 107(2): 663–672. doi: 10.1016/j.cognition.2007.07.015 17765214

[41] Dimoska-Di Marco A., et al. A meta-analysis of response inhibition and Stroop interference control deficits in adults with traumatic brain injury (TBI). *Journal of clinical experimental neuropsychology*. (2011). 33(4): 471–485. doi: 10.1080/13803395.2010.533158 21229434

[42] Leunissen I., et al. Task switching in traumatic brain injury relates to cortico-subcortical integrity. *Human brain mapping*. (2014). 35(5): 2459–2469. doi: 10.1002/hbm.22341 23913872 PMC6869801

[43] Rohatgi, A.J.U.h.a.i.W.a. WebPlotDigitizer user manual version 3.4. (2014). 1–18.

[44] Moola S., et al. Chapter 7: Systematic reviews of etiology and risk. *Joanna Briggs Institute Reviewer’s Manual*. The Joanna Briggs Institute (2017). 5.

[45] Field A.P. and Gillett R. How to do a meta-analysis. *British Journal of Mathematical and Statistical Psychology*. (2010). 63(3): 665–694. doi: 10.1348/000711010X502733 20497626

[46] Hedges L.V. Estimation of a single-effect size: Parametric and non-parametric methods. *Statistical methods for meta-analysis*. (1985).

[47] Cohen J. A power primer. *Psychological bulletin*. (1992). 112(1): 155. doi: 10.1037//0033-2909.112.1.155 19565683

[48] Suurmond R., van Rhee H., and Hak T. Introduction, comparison, and validation of Meta-Essentials: a free and simple tool for meta-analysis. *Research synthesis methods*. (2017). 8(4): 537–553. doi: 10.1002/jrsm.1260 28801932 PMC5725669

[49] Borenstein M., et al. Introduction to meta-analysis. *Chichester*, *WestSussex*: *John Wiley & Sons Ltd*. (2009). 33: 38. doi: 10.1002/9780470743386 29218357

[50] Huedo-Medina T.B., et al. Assessing heterogeneity in meta-analysis: Q statistic or I^2^ index? *Psychological methods*. (2006). 11(2): 193. https://psycnet.apa.org/doi/10.1037/1082-989X.11.2.193. 16784338 10.1037/1082-989X.11.2.193

[51] Moher D., et al. Preferred reporting items for systematic reviews and meta-analyses: the PRISMA statement. *PLoS medicine*. (2009). 6(7): e1000097. doi: 10.1371/journal.pmed.1000097 19621072 PMC2707599

[52] Cremona-Meteyard S.L. and Geffen G.M. Persistent visuospatial attention deficits following mild head injury in Australian rules football players. *Neuropsychologia*. (1994). 32(6): 649–662. doi: 10.1016/0028-3932(94)90026-4 8084421

[53] Heinze H.-J., et al. Parallel and serial visual search after closed head injury: electrophysiological evidence for perceptual dysfunctions. *Neuropsychologia*. (1992). 30(6): 495–514. doi: 10.1016/0028-3932(92)90054-p 1641115

[54] Hill-Jarrett T.G., et al. Visuospatial attention after traumatic brain injury: The role of hemispheric specialization. *Brain injury*. (2015). 29(13–14): 1617–1629. doi: 10.3109/02699052.2015.1075155 26451899

[55] Kim Y.-H., et al. Plasticity of the attentional network after brain injury and cognitive rehabilitation. *Neurorehabilitation* (2009). 23(5): 468–477. doi: 10.1177/1545968308328728 19118131

[56] Malojcic B., et al. Consequences of Mild Traumatic Brain Injury on Information Processing Assessed with Attention and Short-Term Memory Tasks. *Journal of neurotrauma*. (2008). 25(1): 30–37. doi: 10.1089/neu.2007.0384 18355156

[57] Ponsford J. and Kinsella G. Attentional deficits following closed-head injury. *Journal of Clinical and Experimental Neuropsychology*. (1992). 14(5): 822–838. doi: 10.1080/01688639208402865 1474148

[58] Slovarp L., Azuma T., and LaPointe L. The effect of traumatic brain injury on sustained attention and working memory. *Brain injury*. (2012). 26(1): 48–57. doi: 10.3109/02699052.2011.635355 22149444

[59] Stuss D., et al. Reaction time after head injury: fatigue, divided and focused attention, and consistency of performance. *Journal of Neurology*, *Neurosurgery & Psychiatry*. (1989). 52(6): 742–748. doi: 10.1136/jnnp.52.6.742 2746267 PMC1032026

[60] Willmott C., et al. Factors contributing to attentional impairments after traumatic brain injury. *Neuropsychology*. (2009). 23(4): 424. https://psycnet.apa.org/doi/10.1037/a0015058. 19586207 10.1037/a0015058

[61] Ziino C. and Ponsford J. Selective Attention Deficits and Subjective Fatigue Following Traumatic Brain Injury. *Neuropsychology*. (2006). 20(3): 383–390. https://psycnet.apa.org/10.1037/0894-4105.20.3.383. 16719631 10.1037/0894-4105.20.3.383

[62] Halterman C.I., et al. Tracking the recovery of visuospatial attention deficits in mild traumatic brain injury. *Brain*. (2006). 129(3): 747–753. doi: 10.1093/brain/awh705 16330498

[63] van Donkelaar P., Osternig L., and Chou L.-S. Attentional and Biomechanical Deficits Interact After Mild Traumatic Brain Injury. *Exercise and sport sciences reviews*. (2006). 34(2): 77–82. doi: 10.1249/00003677-200604000-00007 16672805

[64] Wu Y., et al. Multiple Component Analysis of Attention Early After Complicated Mild Traumatic Brain Injury: A Prospective Cohort Study. *Journal of Rehabilitation Medicine*. (2020). 52(4): 1–7. doi: 10.2340/16501977-2673 32266412

[65] Register-Mihalik J.K., Littleton A.C., and Guskiewicz K.M. Are divided attention tasks useful in the assessment and management of sport-related concussion? *Neuropsychology review*. (2013). 23(4): 300–313. doi: 10.1007/s11065-013-9238-1 24242888

[66] Ginstfeldt T. and Emanuelson I. An overview of attention deficits after paediatric traumatic brain injury. *Brain Injury*. (2010). 24(10): 1123–1134. doi: 10.3109/02699052.2010.506853 20715886

[67] Lee K. and Choo H. A critical review of selective attention: an interdisciplinary perspective. *Artificial Intelligence Review*. (2013). 40(1): 27–50. doi: 10.1007/s10462-011-9278-y

[68] Brosseau-Lachaine O., et al. Mild traumatic brain injury induces prolonged visual processing deficits in children. *Brain Injury*. (2008). 22(9): 657–668. doi: 10.1080/02699050802203353 18698516

[69] Chang T.T.-L., Ciuffreda K.J., and Kapoor N. Critical flicker frequency and related symptoms in mild traumatic brain injury. *Brain injury*. (2007). 21(10): 1055–1062. doi: 10.1080/02699050701591437 17891568

[70] Patel R., et al. Elevated coherent motion thresholds in mild traumatic brain injury. *Optometry—Journal of the American Optometric Association*. (2011). 82(5): 284–289. doi: 10.1016/j.optm.2010.10.012 21524599

[71] Alnawmasi M.M., et al. The effect of mild traumatic brain injury on the visual processing of global form and motion. *Brain injury*. (2019). 33(10): 1354–1363. doi: 10.1080/02699052.2019.1641842 31317788

[72] McClincy M.P., et al. Recovery from sports concussion in high school and collegiate athletes. *Brain injury*. (2006). 20(1): 33–39. doi: 10.1080/02699050500309817 16403698

[73] McCrory P., et al. Consensus statement on concussion in sport—the 5th international conference on concussion in sport held in Berlin, October 2016. *British journal of sports medicine*. (2017). 51(11): 838–847. doi: 10.1136/bjsports-2017-097699 28446457

[74] Baker J.G., Willer B.S., and Leddy J.J. Integrating neuropsychology services in a multidisciplinary concussion clinic. *The Journal of head trauma rehabilitation*. (2019). 34(6): 419–424. doi: 10.1097/HTR.0000000000000541 31688378

[75] Corwin D.J., et al. Characteristics and outcomes for delayed diagnosis of concussion in pediatric patients presenting to the emergency department. *The Journal of Emergency Medicine*. (2020). 59(6): 795–804. doi: 10.1016/j.jemermed.2020.09.017 33036827 PMC7736137

[76] Williams H., et al. Epidemiology of adults receiving acute inpatient rehabilitation for a primary diagnosis of traumatic brain injury in the United States. *Journal of head trauma rehabilitation*. (2015). 30(2): 122–135. doi: 10.1097/HTR.0000000000000012 24495917

[77] Bogdanova Y., et al. Computerized cognitive rehabilitation of attention and executive function in acquired brain injury: a systematic review. *The Journal of head trauma rehabilitation*. (2016). 31(6): 419. doi: 10.1097/HTR.0000000000000203 26709580 PMC5401713

