# Supplementary material for: Changes in the components of visual attention following traumatic brain injury: A systematic review and meta-analysis
Source: PLoS One. 2022 Jun 9;17(6):e0268951. doi: 10.1371/journal.pone.0268951 (PMC9182329; doi:10.1371/journal.pone.0268951)
Supplement: S1 Fig — Individual effect sizes and combined effect sizes for each component of visual attention (selective A, sustained B, orientation of attention C. (PDF) [file pone.0268951.s001.pdf]

S1 Fig 1.Individual efect sizes and combined efect sizes for each component of visual attention (selective A, sustained B, orientation of attention C

A

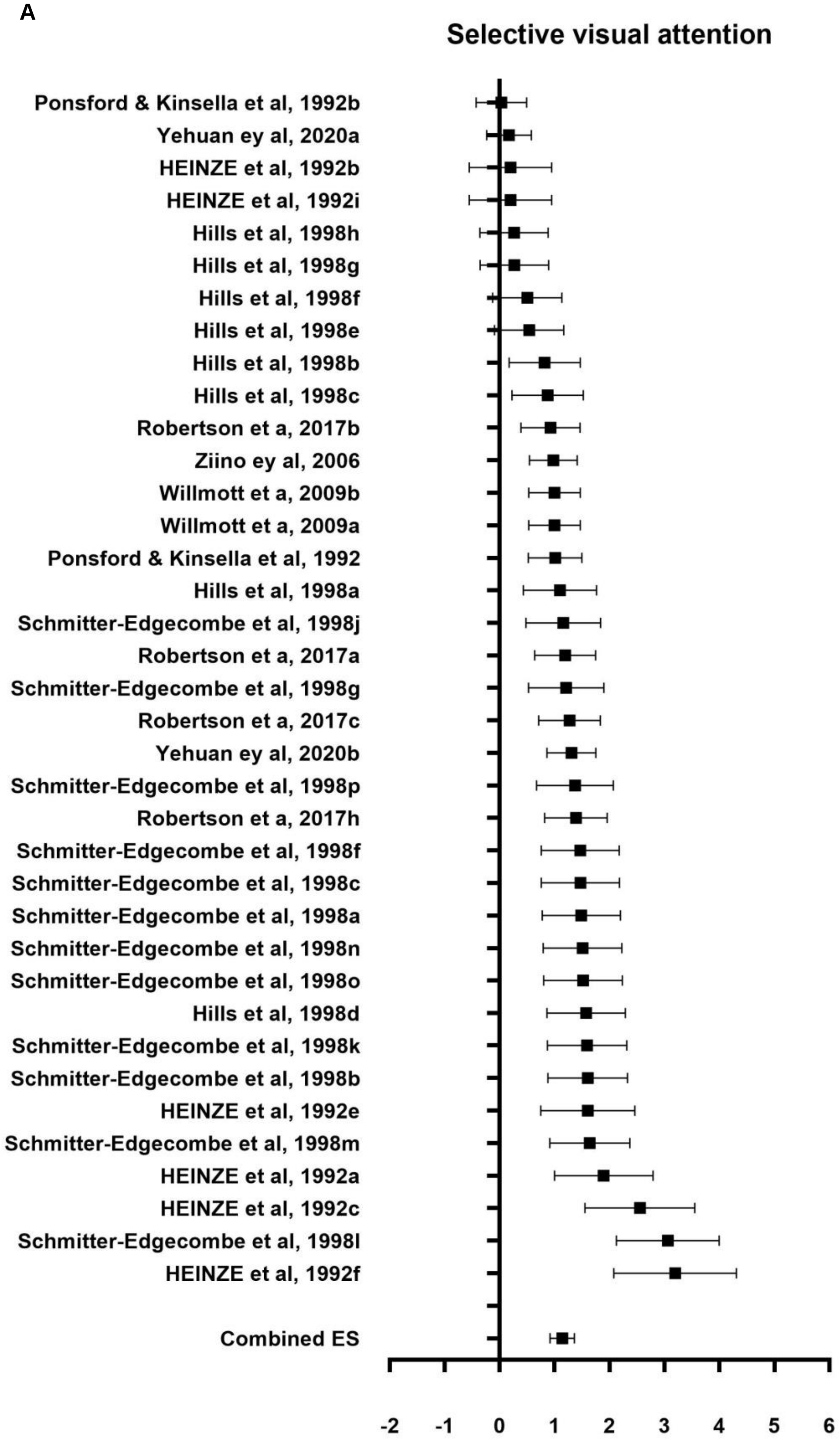

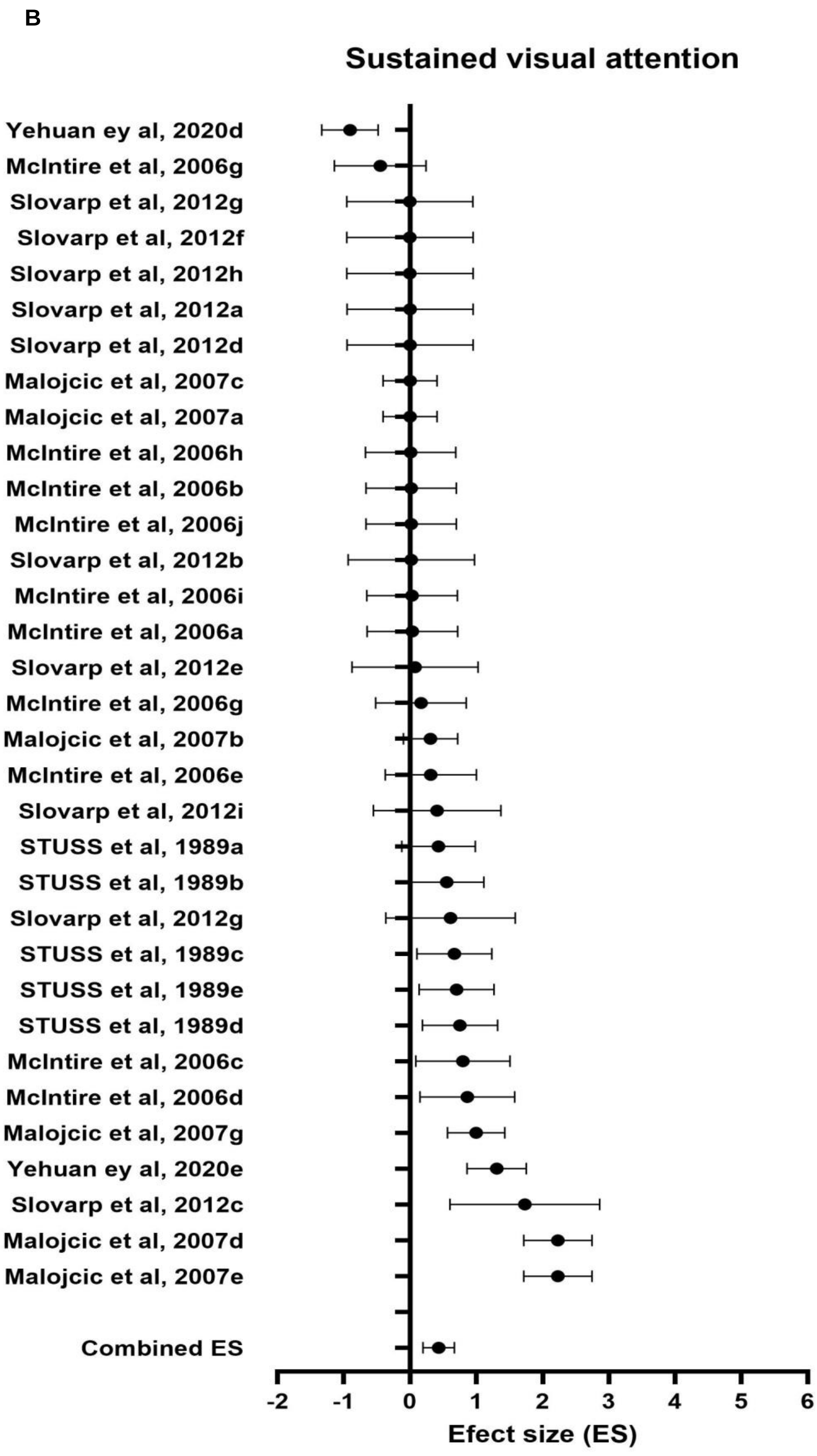
B
Sustained visual attention
Yehuan ey al, 2020d
McIntire et al, 2006g
Slovarp et al, 2012g
Slovarp et al, 2012f
Slovarp et al, 2012h
Slovarp et al, 2012a
Slovarp et al, 2012d
Malojcic et al, 2007c
Malojcic et al, 2007a
McIntire et al, 2006h
McIntire et al, 2006b
McIntire et al, 2006j
Slovarp et al, 2012b
McIntire et al, 2006i
McIntire et al, 2006a
Slovarp et al, 2012e
McIntire et al, 2006g
Malojcic et al, 2007b
McIntire et al, 2006e
Slovarp et al, 2012i
STUSS et al, 1989a
STUSS et al, 1989b
Slovarp et al, 2012g
STUSS et al, 1989c
STUSS et al, 1989e
STUSS et al, 1989d
McIntire et al, 2006c
McIntire et al, 2006d
Malojcic et al, 2007g
Yehuan ey al, 2020e
Slovarp et al, 2012c
Malojcic et al, 2007d
Malojcic et al, 2007e
Combined ES
-2
-1
0
1
2
3
4
5
6
Efect size (ES)

C

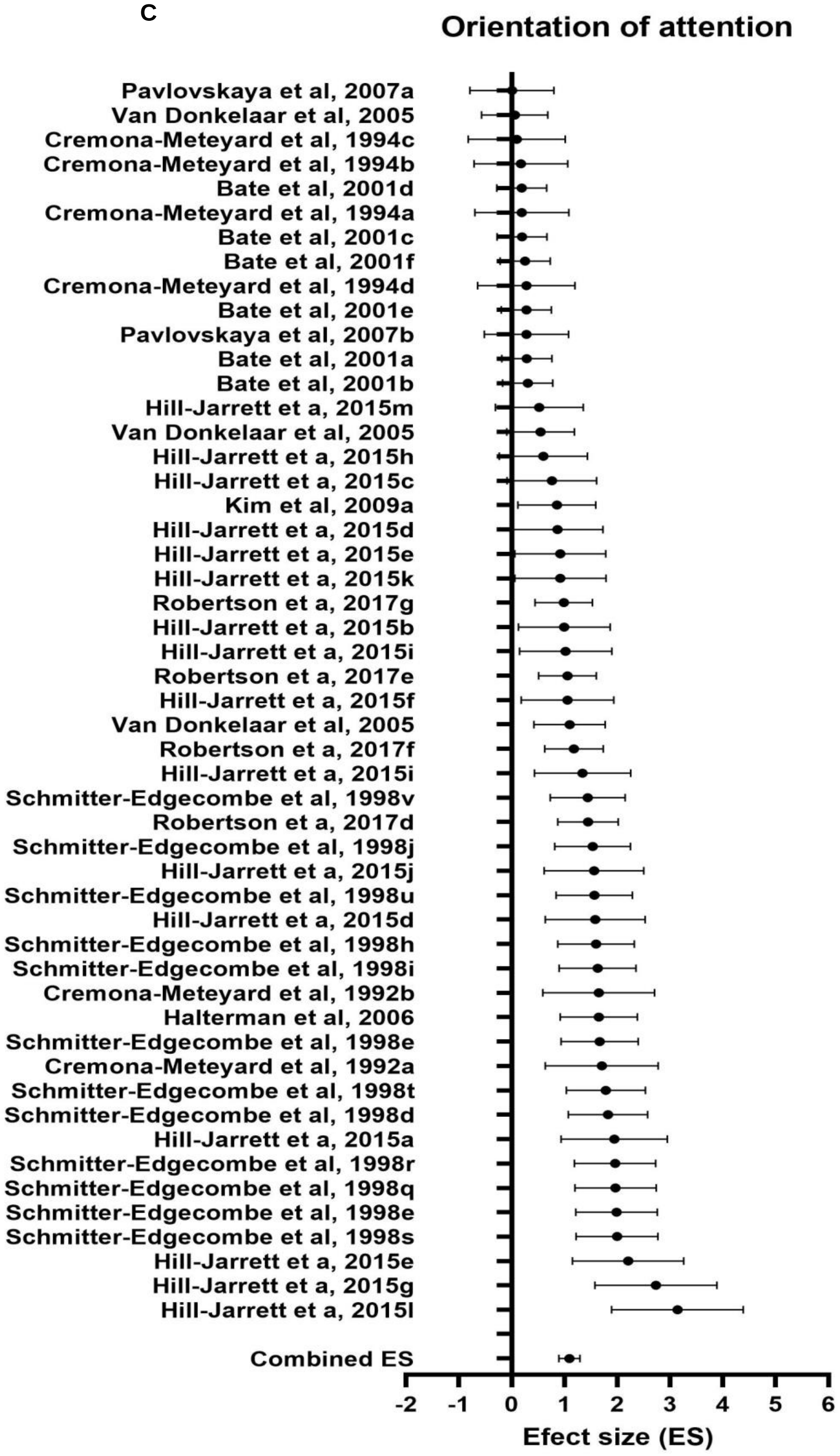
Orientation of attention
Pavlovskaya et al, 2007a
Van Donkelaar et al, 2005
Cremona-Meteyard et al, 1994c
Cremona-Meteyard et al, 1994b
Bate et al, 2001d
Cremona-Meteyard et al, 1994a
Bate et al, 2001c
Bate et al, 2001f
Cremona-Meteyard et al, 1994d
Bate et al, 2001e
Pavlovskaya et al, 2007b
Bate et al, 2001a
Bate et al, 2001b
Hill-Jarrett et a, 2015m
Van Donkelaar et al, 2005
Hill-Jarrett et a, 2015h
Hill-Jarrett et a, 2015c
Kim et al, 2009a
Hill-Jarrett et a, 2015d
Hill-Jarrett et a, 2015e
Hill-Jarrett et a, 2015k
Robertson et a, 2017g
Hill-Jarrett et a, 2015b
Hill-Jarrett et a, 2015i
Robertson et a, 2017e
Hill-Jarrett et a, 2015f
Van Donkelaar et al, 2005
Robertson et a, 2017f
Hill-Jarrett et a, 2015i
Schmitter-Edgecombe et al, 1998v
Robertson et a, 2017d
Schmitter-Edgecombe et al, 1998j
Hill-Jarrett et a, 2015j
Schmitter-Edgecombe et al, 1998u
Hill-Jarrett et a, 2015d
Schmitter-Edgecombe et al, 1998h
Schmitter-Edgecombe et al, 1998i
Cremona-Meteyard et al, 1992b
Halterman et al, 2006
Schmitter-Edgecombe et al, 1998e
Cremona-Meteyard et al, 1992a
Schmitter-Edgecombe et al, 1998t
Schmitter-Edgecombe et al, 1998d
Hill-Jarrett et a, 2015a
Schmitter-Edgecombe et al, 1998r
Schmitter-Edgecombe et al, 1998q
Schmitter-Edgecombe et al, 1998e
Schmitter-Edgecombe et al, 1998s
Hill-Jarrett et a, 2015e
Hill-Jarrett et a, 2015g
Hill-Jarrett et a, 2015l
Combined ES
-2 -1 0 1 2 3 4 5 6
Efect size (ES)
